# The optimal first-line treatment for patients with left-sided RAS wild-type metastatic colorectal cancer: Double-drug regimen or triple-drug regimen therapy

**DOI:** 10.3389/fphar.2022.1015510

**Published:** 2022-09-30

**Authors:** Changjing Cai, Qingqing Luo, Yihan Liu, Yinghui Peng, Xiangyang Zhang, Zhaohui Jiang, Ziyang Feng, Yaru Qi, Yan Gao, Yongting Liu, Ping Liu, Yihong Chen, Cao Guo, Hong Shen, Shan Zeng, Ying Han

**Affiliations:** ^1^ Department of Oncology, Xiangya Hospital, Central South University, Changsha, China; ^2^ National Clinical Research Center for Geriatric Disorders, Xiangya Hospital, Central South University, Changsha, China; ^3^ Department of Oncology, Hunan Provincial People’s Hospital, The First Affiliated Hospital of Hunan Normal University, Changsha, China

**Keywords:** colorectal cancer, cetuximab, panitumumab, bevacizumab, targeted therapy

## Abstract

There are many treatments for metastatic colorectal cancer (mCRC). Among them, uncertainty remains especially concerning the clinical benefit of different regimens for left-sided RAS wild-type (WT) mCRC in the triple-drug therapy era. No studies have been conducted to answer this critical clinical issue. We performed a comprehensive analysis of published data and real-world data. First, we conducted analyses of the published trials to show the landscape of efficacy and safety in the treatments of left-sided RAS WT mCRC. Then, we initiated a multicenter real-world study as the validation dataset. This study included six published randomized controlled trials (RCTs) and a total of 1925 patients. The double-drug regimen plus cetuximab/panitumumab (D + C/P) achieved the longest overall survival (OS) in patients with left-sided mCRC (HR = 0.74, 95%CI: 0.57–0.98), while triple-drug regimen with bevacizumab (T + B, HR = 1.1, 95%CI: 0.63–2.0), compared with double-drug with bevacizumab (D + B). The D + C/P had the highest overall response rate (ORR) in patients with left-sided mCRC (OR = 1.8, 95%CI: 0.89–3.8), while T + B (OR = 1.8, 95%CI: 0.70–4.8), compared with D + B. The multicenter real-world cohort showed the double-drug regimen plus cetuximab had longer progression-free survival (PFS) in left-sided mCRC patients than the triple-drug regimen with bevacizumab. The safety analysis showed the incidence of the adverse events (grade≥3) in the triple-drug therapy plus bevacizumab was higher than that in the double-drug therapy plus cetuximab/panitumumab. This work demonstrates the ranking of three regimens for therapeutic efficacy and safety in patients with left-sided RAS WT mCRC. The double-drug regimen plus cetuximab/panitumumab appears more effective and safer than double-drug and triple-drug based regimens with bevacizumab. Further trials and cohort analyses on this topic would increase confidence in these results.

## Introduction

Colorectal cancer (CRC), accounting for about 10% of cancer-related mortality worldwide, is the third most common cancer, with an estimated 1.8 million new cases globally in 2018 (Bray et al., 2018). The 5-year relative survival is 71–90% for locoregional disease and 14% for distant-stage disease in the United States of America (United States) (Nitsche et al., 2011). The backbone of first-line chemotherapies for metastatic CRC (mCRC) consists of fluoropyrimidine (FP) [intravenous (IV) 5-fluorouracil (5-FU) or oral capecitabine] in various combinations and schedules (Tournigand et al., 2004; Haller et al., 2005; Sargent et al., 2011; Van Cutsem et al., 2016; NCCN Clinical Practice Guidelines in oncology, 2020; NCCN Clinical Practice Guidelines in oncology, 2021). The current guidelines advocate the combinations of the FP with oxaliplatin (FOLFOX: 5-FU, leucovorin, and oxaliplatin) or irinotecan (FOLFIRI: 5-FU, leucovorin, and irinotecan) (Van Cutsem et al., 2016; NCCN Clinical Practice Guidelines in oncology, 2020; NCCN Clinical Practice Guidelines in oncology, 2021). Recently, new data have emerged on using triple-drug chemotherapy with 5-FU, leucovorin, oxaliplatin, and irinotecan, named FOLFOXIRI (Assenat et al., 2011; Fornaro et al., 2013; Loupakis et al., 2014; Cremolini et al., 2018).

Targeted therapy using monoclonal antibodies binding to the epidermal growth factor receptor (EGFR) or the vascular endothelial growth factor (VEGF) is currently considered to be the standard therapy for the first-line treatment of mCRC (Nordlinger et al., 2009). A large meta-analysis showed that left-sided colon cancer is associated with a 19% reduced risk of death than right-sided colon cancer, independently of TNM stage, race, adjuvant chemotherapy, year of the studies, and the number of enrollment (Petrelli et al., 2017). A previous meta-analysis compared the efficacy and safety outcomes of bevacizumab, panitumumab, or cetuximab with chemotherapy in mCRC. It showed that cetuximab, closely followed by panitumumab, was the most effective treatment in left-sided RAS wild-type (WT) mCRC, and bevacizumab was more effective in right-sided mCRC (Wu et al., 2020). Still, in the previous works, the differences between triple-drug and double-drug backbone chemotherapy were not considered. Thus, uncertainty remains concerning the clinical benefit of different regimens for left-sided RAS WT mCRC in the triple-drug therapy era.

Therefore, we performed a comprehensive analysis of the published data and real-world data. First, we conducted a network meta-analysis (NMA) analysis of the published trials to show the landscape of the treatments in left-sided RAS WT mCRC. Then, according to the NMA results, we initiated a real-world multicenter study as the validation dataset. We hope this study can guide clinicians in individual decision-making in treating left-sided RAS WT mCRC.

## Methods

### Part 1: NMA in the published trials

The study was registered in PROSPERO (CRD42022329992).

### Literature search

The present study was carried out according to the Preferred Reporting Items for Systematic Reviews and Meta-Analyses (PRISMA) guidelines (Selcuk, 2019). The meta-analysis was designed based on the PICOS principle (Aslam and Emmanuel, 2010). PubMed, Embase, and the Cochrane Library were searched for potentially eligible studies published from inception up to May 2021 using the MeSH terms of “colorectal cancer”, “chemotherapy”, “molecular targeted therapy”, “cetuximab”, “panitumumab”, and “bevacizumab”, as well as relevant keywords. Then, two investigators screened the retrieved records and retrieved full-text articles according to the inclusion and exclusion criteria. Disagreements were solved by discussion until a consensus was reached. The reference lists of the retrieved articles were also searched for additional potentially eligible studies. If multiple papers reported the same trial, only the most recent one was kept.

### Eligibility criteria

The inclusion criteria were 1) patients: adult (>18 years) patients with RAS WT mCRC, 2) interventions: first-line double-drug (FOLFOX or FOLFIRI) chemotherapy combined with cetuximab, panitumumab, or bevacizumab or first-line triple-drug therapy (FOLFOXIRI) with cetuximab, panitumumab, or bevacizumab, 3) outcome: overall survival (OS, ORR, or PFS, 4) study design: randomized controlled trials (RCTs), 5) published in English, and 6) the full text was available. The exclusion criteria were 1) data about left-sided mCRC were not reported, 2) insufficient data for meta-analysis, or 3) non-human studies.

### Study selection

Supplementary Figure S1 presents the study flowchart. The initial search yielded 2,494 records, and three additional records were identified through the manual search of reference lists. After removing 606 duplicates, 1891 records were screened. We excluded 69 meta-analyses, 211 protocol publications, 86 conference abstracts, 20 editorials, 577 reviews, and 97 notes/reports/surveys/letters. 11 publications cannot be identified, and 23 full-text cannot be retrieved. For the remaining 797 articles, we excluded 380 articles that were not relevant to the present study hypothesis, 76 articles that were irrelevant with RAS wild-type metastatic colorectal cancer, 271 articles irrelevant with first-line double-drug/triple-drug chemotherapy regimen combined with cetuximab, panitumumab or bevacizumab, four articles not reporting left-sided primary tumor data, 23 articles not reporting OS, ORR, and PFS, 16 articles were the update of previous studies and 21 non-English publications. Finally, six RCTs were selected for the NMA.

### Data extraction

Study characteristics (name of the trial, start date of the trial, country where the trial was performed, experimental arm, control arm, and sample size), patients’ characteristics (sex, age, and mutations), the primary outcome (OS with follow-up duration), and secondary outcomes (ORR and PFS with follow-up duration) were extracted by two investigators following a standardized form. Discrepancies in the assessment were solved by discussion. All the safety data (Adverse events (AEs)) of the six RCTs have been included.

### Quality of the evidence

The level of the evidence of all articles was assessed independently by two investigators according to the Version two of the Cochrane risk-of-bias assessment tool (ROB 2) for evaluating RCTs (Sterne et al., 2019). Discrepancies in the assessment were resolved through discussion until a consensus was reached.

### Geometry of the network

A network plot was drawn to describe and present the geometry of the treatment network of comparisons across trials to ensure that an NMA was feasible. Trials were excluded if they were not connected to treatments. Network geometry used nodes to represent different interventions and edges to represent the head-to-head comparisons (Rouse et al., 2017; Higgins et al., 2020).

### Part 2: The real-world multicenter study in the validation dataset

The colorectal cancer cases in the validation retrospective cohort (n = 92) were collected between April 2013 and May 2022 in Xiangya hospital and Hunan People’s Hospital. Informed consent was obtained from the recruited patients, and the study protocols were approved by the Ethics Committees of the Xiangya hospital. Included patients meet the following criteria: 1) pathological diagnosis of colorectal cancer; 2) clinical diagnosis of advanced; 3) receipt of first-line cetuximab + chemotherapy with FOLFOX or FOLFIRI; Bevacizumab + FOLFOXIRI; and 4) no severe or fatal diseases. Exclusion criteria were as follows: less than four cycles of chemotherapy.

The follow-up (terminated on 30 April 2022) period was defined as the interval between the date of random assignment and that of the patient’s death or the last follow-up. The primary study endpoints were PFS, defined as the time from the initial therapy date to tumor progression, death from any cause, or the last follow-up before the initiation of second-line therapy. Every 2 months, the response to treatment was evaluated by computed tomography scans according to the Response Evaluation Criteria in Solid Tumors (RECIST, version 1.1). (Supplementary Table S2).

### Statistical analysis

All statistical analyses were conducted using R version 4.0.2 (The R Project for Statistical Computing, www.r-project.org). NMAs were performed using the gemtc 0.8-8 library, which is based on the Bayesian probability framework. Because significant heterogeneity among studies was expected due to differences in methodology, drugs, and patient populations, the random-effects model was used for all analyses. OS and PFS were reported as hazard ratios (HRs) with corresponding 95% confidence intervals (CIs). The ORR was reported as odds ratios (ORs) with corresponding 95% CIs. Forest plots were used to show each treatment’s effect sizes (HR and OR) compared to double-drug therapy with bevacizumab. All network models were run for a minimum of 100,000 iterations to ensure convergence. A subgroup analysis was performed in patients with both RAS/BRAF WT. The probability for each treatment was determined to be the best, second best, third best, and so forth options. The results were plotted in a rankogram. Two-sided *p*-values <0.05 were considered statistically significant. AEs rates and their corresponding 95% confidence intervals were estimated using both a fixed-effects model and a random-effects model. The log-rank test and Kaplan-Meier plotter had been used in the survival analysis of real-world cohort.

## Results

### Study characteristics and quality assessment of NMA analysis

The characteristics of included studies are presented in Supplementary Table S1. Two studies were from North America (Venook et al., 2017; Hurwitz et al., 2019), two were from Italy (Cremolini et al., 2015; Cremolini et al., 2017), and two were performed across at least two continents (Heinemann et al., 2014; Rivera et al., 2017). The total number of patients with left-sided mCRC was 1925. Two trials compared two double-drug regimens (Heinemann et al., 2014; Venook et al., 2017), and four compared triple-drug vs. double-drug regimens (Cremolini et al., 2015; Cremolini et al., 2017; Rivera et al., 2017; Hurwitz et al., 2019). The median follow-up ranged from 22.2 to 48.1 months. Four studies reported the BRAF WT status. Quality assessment of the included RCTs is presented in Supplementary Figure S2 according to ROB 2. Only one RCT had a low risk of bias for all items (Cremolini et al., 2017). All other trials had at least one item with an unclear risk of bias (Heinemann et al., 2014; Cremolini et al., 2015; Rivera et al., 2017; Venook et al., 2017; Hurwitz et al., 2019).

### OS of NMA analysis

**FIGURE 1:**
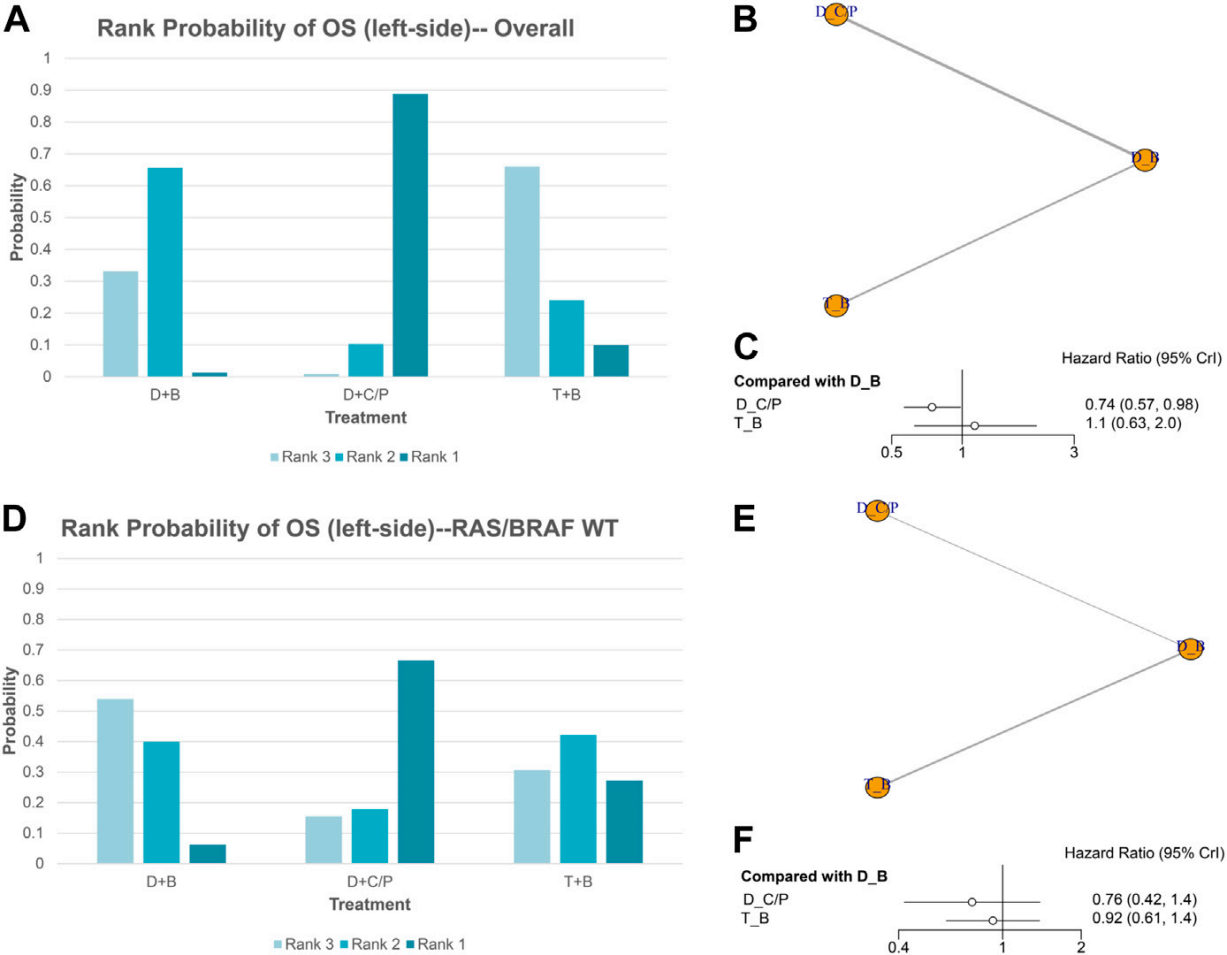
Overall survival (OS) in overall and the subgroup of RAS/BRAF wild-type patients. Overall: **(A)** The rank probability of the benefits of the treatments. **(B)** Network of eligible comparisons for network meta-analysis. **(C)** Forest plot of comparisons to double-drug regimen plus bevacizumab. RAS/BRAF WT: **(D)** The rank probability of the benefits of the treatments. **(E)** Network of eligible comparisons for network meta-analysis. **(F)** Forest plot of comparisons to double-drug regimen plus bevacizumab. (*N* = 1925, B: bevacizumab; C: cetuximab; D: double-drug; P: panitumumab; T: triple-drug.)

Figure 1 shows the network of the eligible comparisons for OS of the NMA. Five trials could be included in the analysis of the OS. According to the rank probability plot, compared with the double-drug therapy with bevacizumab, the double-drug regimen plus cetuximab/panitumumab achieved the longest OS in patients with left-sided mCRC (HR = 0.74, 95%CI: 0.57–0.98, *p* < 0.05) (Figures 1A–C; Table 1). There was no significant difference between the double-drug therapy with bevacizumab and the triple-drug therapy with bevacizumab (HR = 1.1, 95%CI: 0.63–2.0). In the subgroup analysis of left-sided tumors with RAS/BRAF WT, there were no significant differences among the double-drug therapy with bevacizumab, the double-drug therapy with cetuximab/panitumumab, and the triple-drug therapy with bevacizumab. From the rank probability plot, the double-drug regimen plus cetuximab/panitumumab had the highest OS in patients with left-sided mCRC and wild type RAS and BRAF (OR = 0.76, 95%CI: 0.42–1.40, compared with the double-drug regimen with bevacizumab), followed by the triple-drug regimen with bevacizumab (OR = 0.92, 95%CI: 0.61–1.40, compared with the double-drug regimen with bevacizumab) (Figures 1D–F).

**TABLE 1:** Synthesis of odds ratios according to the network meta-analysis (ORR, OS, and PFS).

	D + B	D + C/P	T + B
**OS**			
D + B	D + B	0.745 (0.568, 0.981)	1.136 (0.624, 2.073)
D + C/P	1.342 (1.019, 1.761)*	D + C/P	1.524 (0.789, 2.958)
T + B	0.88 (0.482, 1.601)	0.656 (0.338, 1.268)	T + B
**ORR**			
D + B	D + B	1.791 (0.883, 3.805)	1.79 (0.701, 4.735)
D + C/P	0.558 (0.263, 1.133)	D + C/P	0.999 (0.306, 3.308)
T + B	0.559 (0.211, 1.427)	1.001 (0.302, 3.27)	T + B
**PFS**			
D + B	D + B	0.852 (0.613, 1.143)	0.801 (0.518, 1.272)
D + C/P	1.174 (0.875, 1.632)	D + C/P	0.94 (0.563, 1.674)
T + B	1.249 (0.786, 1.93)	1.064 (0.597, 1.777)	T + B

B, bevacizumab; C, cetuximab; D, double-drug; P, panitumumab; T, triple-drug.

OS, overall survival; ORR, overall response rate; PFS, progression-free survival. *p < 0.05.

### ORR of NMA analysis

**FIGURE 2:**
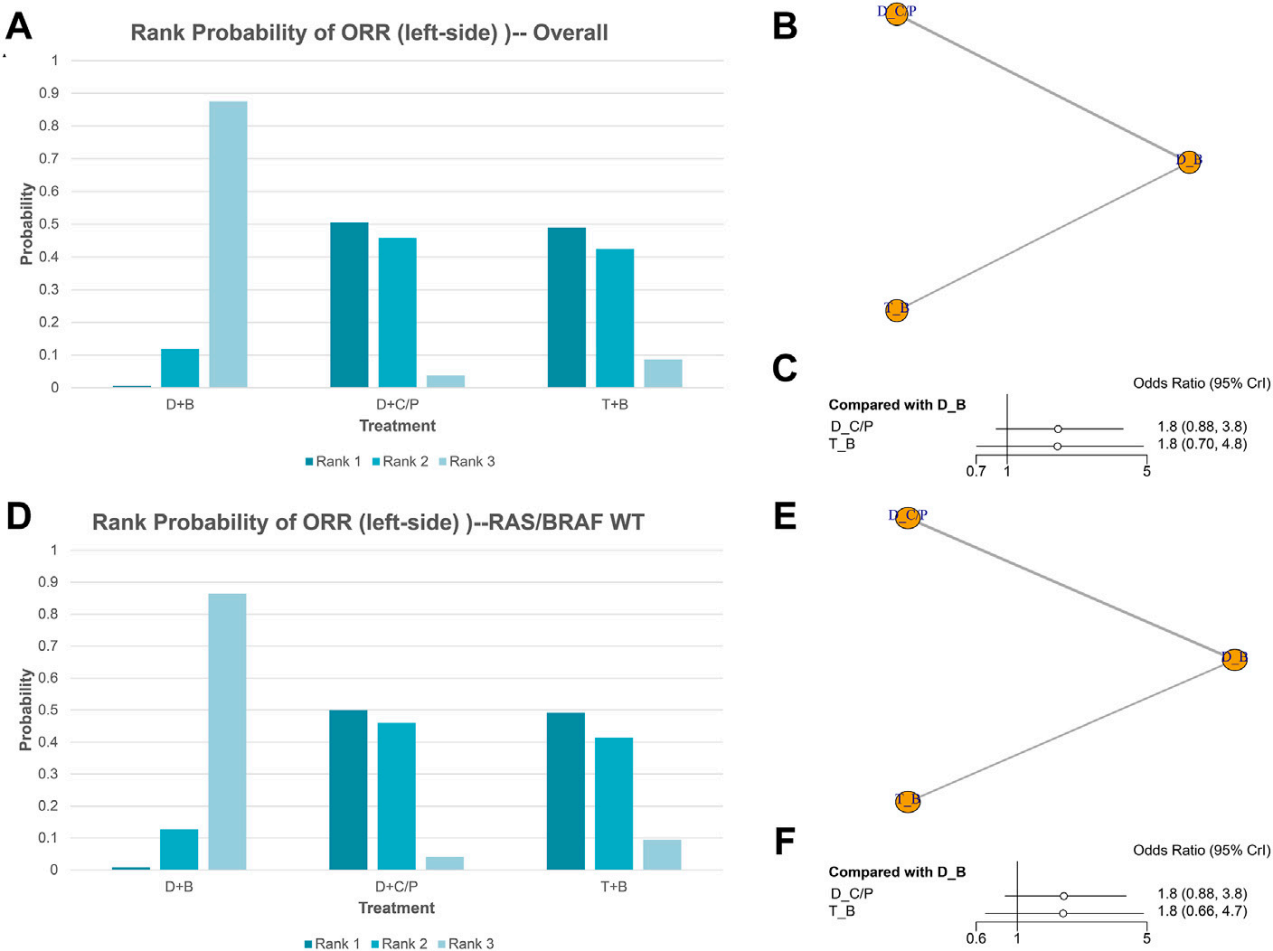
Overall response rate (ORR) in overall and the subgroup of RAS/BRAF wild-type patients. Overall: **(A)** The rank probability of the benefits of the treatments. **(B)** Network of eligible comparisons for network meta-analysis. **(C)** Forest plot of comparisons to double-drug regimen plus bevacizumab. RAS/BRAF WT: **(D)** The rank probability of the benefits of the treatments. **(E)** Network of eligible comparisons for network meta-analysis. **(F)** Forest plot of comparisons to double-drug regimen plus bevacizumab. (*N* = 1925, B: bevacizumab; C: cetuximab; D: double-drug; P: panitumumab; T: triple-drug).

Figure 2 shows the network of the eligible comparisons for ORR of the NMA. Five trials were included for ORR. There were no significant differences among the double-drug therapy with bevacizumab, the double-drug therapy with cetuximab/panitumumab, and the triple-drug therapy with bevacizumab. Still, from the rank probability plot, the double-drug regimen plus cetuximab/panitumumab had the highest ORR in patients with left-sided mCRC (OR = 1.8, 95%CI: 0.89–3.8, compared with the double-drug regimen with bevacizumab), followed by the triple-drug regimen with bevacizumab (OR = 1.8, 95%CI: 0.70–4.8, compared with the double-drug regimen with bevacizumab) (Figures 2A–C; Table 1). In the subgroup analysis of left-sided CRC with RAS/BRAF WT, there were no significant differences among the double-drug therapy with bevacizumab, the double-drug therapy with cetuximab/panitumumab, and the triple-drug therapy with bevacizumab. From the rank probability plot, the double-drug regimen plus cetuximab/panitumumab had the highest ORR in patients with left-sided mCRC and RAS/BRAF WT (OR = 1.8, 95%CI: 0.88–3.80, compared with the double-drug regimen with bevacizumab), followed by the triple-drug regimen with bevacizumab (OR = 1.8, 95%CI: 0.66–4.70, compared with the double-drug regimen with bevacizumab) (Figures 2D–F).

### PFS of NMA analysis

**FIGURE 3:**
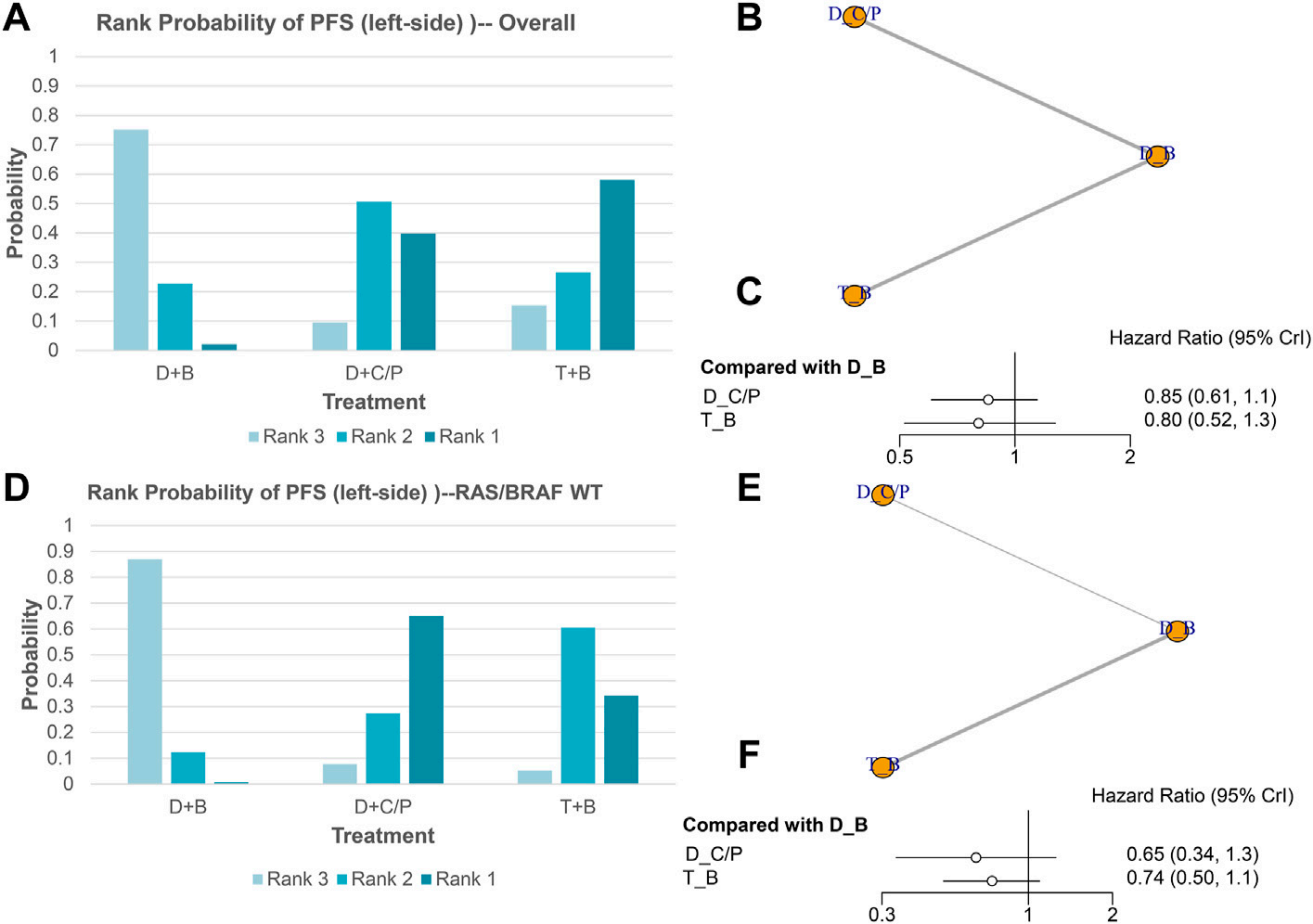
Progression-free survival (PFS) in overall and the subgroup of RAS/BRAF wild-type patients. Overall: **(A)** The rank probability of the benefits of the treatments. **(B)** Network of eligible comparisons for network meta-analysis. **(C)** Forest plot of comparisons to double-drug regimen plus bevacizumab. RAS/BRAF WT: **(D)** The rank probability of the benefits of the treatments. **(E)** Network of eligible comparisons for network meta-analysis. **(F)** Forest plot of comparisons to double-drug regimen plus bevacizumab. (N = 1925, B: bevacizumab; C: cetuximab; D: double-drug; P: panitumumab; T: triple-drug).

Figure 3 shows the network of eligible comparisons for PFS of the network meta-analysis. All six trials were included for the PFS NMA. There were no significant differences among the double-drug therapy with bevacizumab, the double-drug therapy with cetuximab/panitumumab, and the triple-drug therapy with bevacizumab. Still, from the rank probability plot, FOLFOXIRI plus bevacizumab appeared to have similar PFS in patients with left-sided mCRC (HR = 0.80, 95%CI: 0.52–1.3), compared with the double-drug therapy with cetuximab/panitumumab (HR = 0.85, 95%CI: 0.61–1.2) (Figures 3A–C; Table 1). Similar results were observed in the subgroup analysis of RAS/BRAF WT tumors. The double-drug regimen plus cetuximab/panitumumab had the highest ORR in patients with left-sided mCRC and RAS/BRAF WT (OR = 0.65, 95%CI: 0.34–1.30, compared with the double-drug regimen with bevacizumab), followed by triple-drug regimen with bevacizumab (OR = 0.74, 95%CI: 0.50–1.10, compared with the double-drug regimen with bevacizumab) (Figures 3D–F).

### Safety analyses of published trials

**FIGURE 4:**
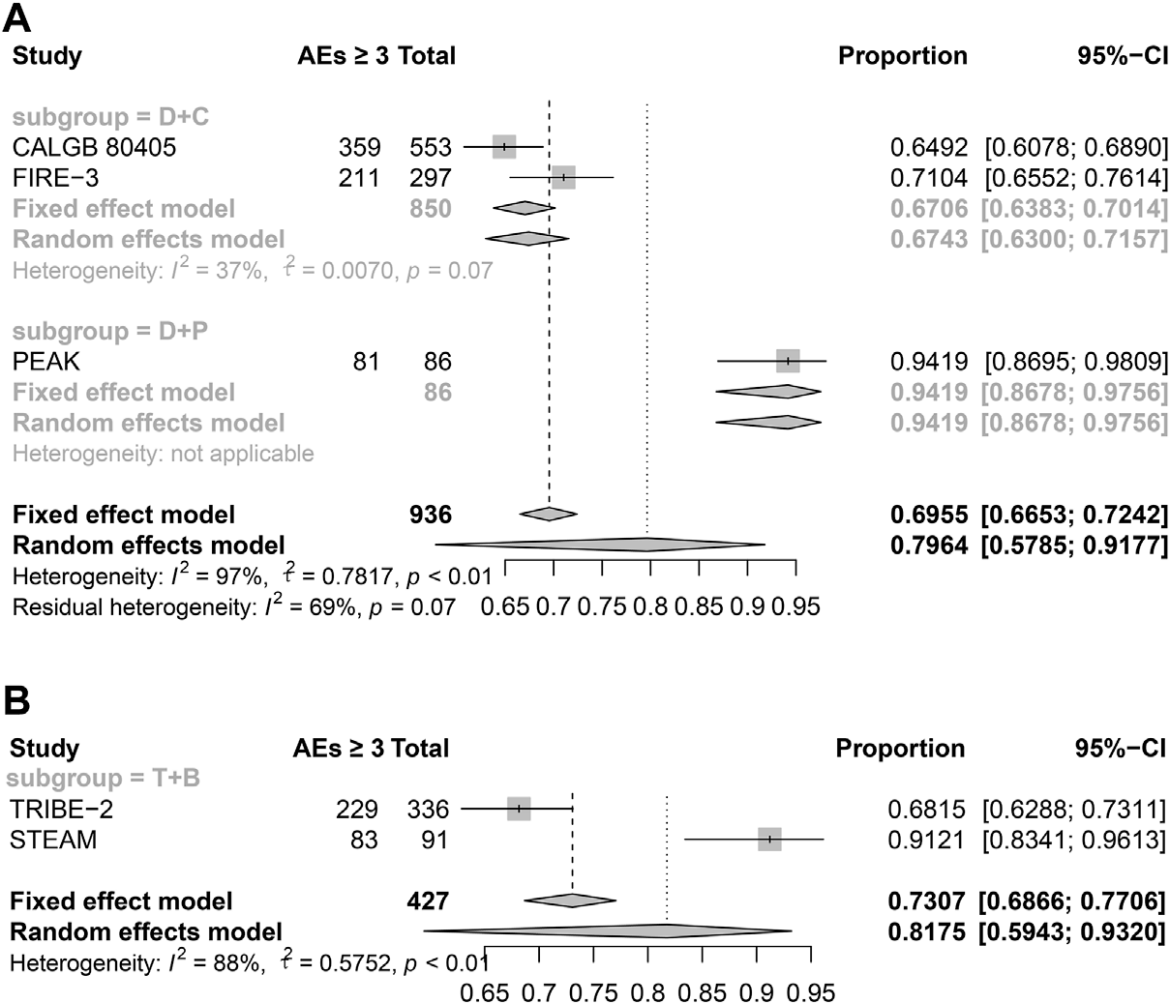
The AEs of different treatments (Grade ≥3). **(A)** Double-drug regimen plus cetuximab/panitumumab. **(B)** Triple-drug regimen plus bevacizumab. (N = 1,363, B: bevacizumab; C: cetuximab; D: double-drug; P: panitumumab; T: triple-drug).

According to the NMA results, it is surprising that the double-drug regimen plus cetuximab/panitumumab showed better efficacy than the triple-drug regimen with bevacizumab. To investigate drug safety, we have included the AEs over grade 3. The results showed the triple-drug regimen with bevacizumab has a high incidence (81.75%), while the double-drug regimen plus cetuximab/panitumumab is 79.64%. What’s more, in the subgroup analyses, the double-drug regimen plus cetuximab has the lowest incidence among others (67.43%) (Figure 4). Haemato-toxicity, fatigue, and diarrhea are the most common AEs in both the triple-drug regimen and the double-drug regimen. However, the triple-drug regimen with bevacizumab carried higher incidences than that in the double-drug regimen. Skin reaction and sensory neuropathy were observed in the double-drug regimen plus cetuximab/panitumumab, while hypertension and hypokalemia were identified in the triple-drug regimen with bevacizumab. (Table 2).

**TABLE 2:** The safety of different treatments.

Study	Subgroup	N	Grade≥3	AEs with grade ≥3 (≥10%)
CALGB 80405	D + C	553	359	Haematotoxicity (32%); Fatigue (10%); Diarrhoea (11%); Sensory neuropathy (13%)
PEAK	D + P	86	81	Skin reaction (15%)
FIRE-3	D + C	297	211	Haematotoxicity (25%); Skin reaction (26%); Diarrhoea (11%)
TRIBE-2	T + B	336	229	Haematotoxicity (50%); Diarrhoea (17%)
STEAM	T + B	91	83	Haematotoxicity (57%); Diarrhoea (22%); Hypertension (22%); Fatigue (12%); Hypokalemia (11%)

B, bevacizumab; C, cetuximab; D, double-drug; P, panitumumab; T, triple-drug.

### Real-world multicenter study validation

To validate the results of the NMA analysis, we initiated a multicenter real-world retrospective study. There are 92 patients included in our cohort. Since there were not enough patients who received the triple-drug regimen with bevacizumab treatment with left-sided RAS WT mCRC, all the RAS WT mCRC patients were included.

**FIGURE 5:**
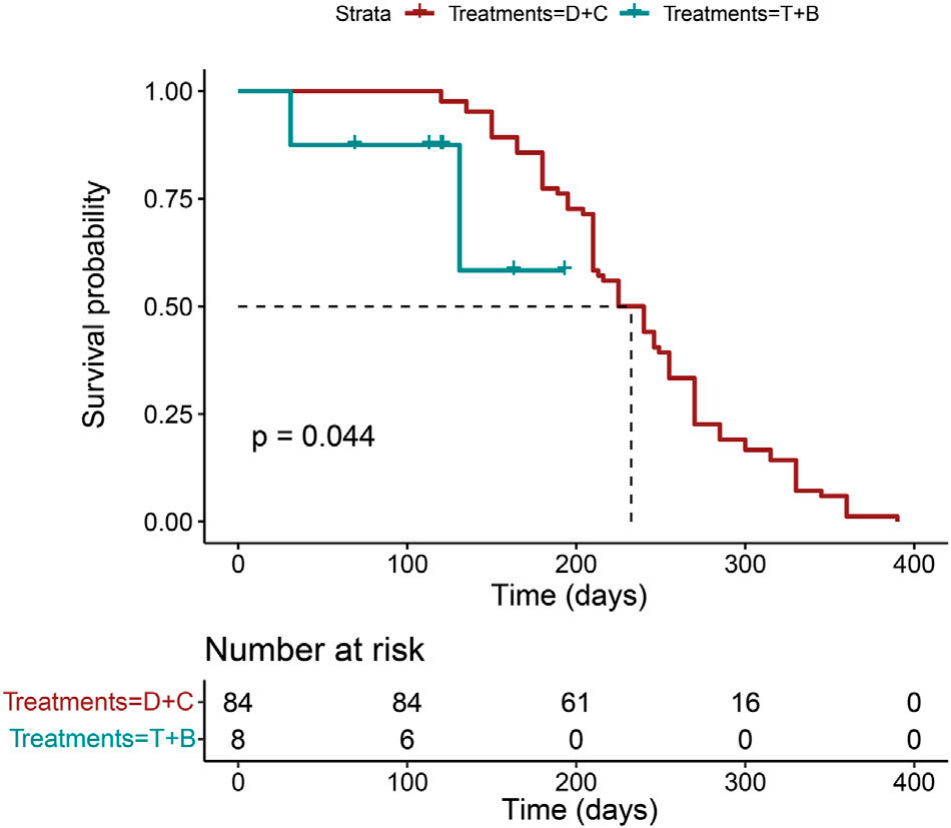
The PFS analysis in the real-world multicenter cohort. (N = 92, *p* = 0.044, log-rank test. B: bevacizumab; C: cetuximab; D: double-drug; P: panitumumab; T: triple-drug).

At the same time, the OS time in the triple-drug regimen with the bevacizumab group has not reached yet, so we chose PFS as the primary outcome. Our results showed the double-drug regimen plus cetuximab had a longer PFS time than the triple-drug regimen with bevacizumab (*p* = 0.04, log-rank), and the HR is 3.93 (95%CI: 0.893–17.3), which is consistent with the results of NMA analysis. (Figure 5).

## Discussion

Uncertainty remains concerning the clinical benefit of different regimens for left-sided RAS WT mCRC in the triple-drug therapy era. Therefore, this NMA aimed to explore the comparative efficacy of different first-line regimens in treating left-sided RAS WT mCRC. The results suggest that a double-drug regimen plus cetuximab/panitumumab appears more effective than double-drug- and triple-drug-based regimens with bevacizumab as first-line therapy for left-sided RAS WT mCRC, and the results had been validated by real-world cohort partly. The safety analysis showed double-drug regimen plus cetuximab/panitumumab is safer than triple-drug regimen with bevacizumab.

This Bayesian network meta-analysis systematically reviewed the short-term (ORR) and long-term (PFS and OS) outcomes of double-drug- or triple-drug-based targeted therapy regimens for left-sided RAS WT mCRC. In the present meta-analysis, for left-sided RAS WT mCRC, the double-drug regimen plus cetuximab/panitumumab appears to be more beneficial than the other two options regarding ORR and OS. A previous meta-analysis showed that cetuximab and panitumumab were more effective in left-sided RAS WT mCRC (Wu et al., 2020). As supported by the previous meta-analysis, cetuximab/panitumumab plus the double-drug regimen was better than bevacizumab plus the double-drug regimen. Still, the previous meta-analysis did not consider the triple-drug regimen with bevacizumab. Thus, triple-drug therapy emerged as an appealing new option for mCRC (Assenat et al., 2011; Fornaro et al., 2013; Loupakis et al., 2014; Cremolini et al., 2018). Notably, the present meta-analysis does not show that the triple-drug regimen fares less well than the double-drug therapy, but that the double-drug therapy with cetuximab/panitumumab achieves better benefits than the triple-drug therapy with bevacizumab. Unfortunately, no study that combined the triple-drug therapy with cetuximab/panitumumab could be included. Therefore, the present NMA supports the inclusion of cetuximab/panitumumab in the regimen for left-sided RAS WT mCRC. Since a meta-analysis showed that the triple-drug regimen achieved better efficacy than the double-drug regimens (Marques et al., 2017), future studies should consider comparing triple-drug therapy with cetuximab/panitumumab.

The V600E BRAF mutation confers resistance to panitumumab or cetuximab (Di Nicolantonio et al., 2008; NCCN Clinical Practice Guidelines in oncology, 2020; NCCN Clinical Practice Guidelines in oncology, 2021). In the overall analysis (i.e., RAS WT but including both BRAF WT and mutants), the double-drug therapy with cetuximab/panitumumab had a significant advantage over the double-drug therapy with bevacizumab. Still, this advantage was lost when analyzing only the RAS/BRAF WT patients, which is surprising since BRAF WT mCRC should respond better to panitumumab or cetuximab according to previous studies (Di Nicolantonio et al., 2008; NCCN Clinical Practice Guidelines in oncology, 2020; NCCN Clinical Practice Guidelines in oncology, 2021). This discrepancy might be due to the small number of studies included in the subgroup analysis. Additional studies are necessary to examine this point.

This study held some practical applications. Rather than just grouping various treatments by the targeted agents or chemotherapy, this NMA assessed three common regimens individually and compared all the major efficacy outcomes regarding the sidedness of primary tumor location. Bayesian NMA allows the comparison of therapies indirectly when no head-to-head trials are available, and, in this study, it acquired more sufficient effect estimates by combining direct and indirect comparisons. What’s more, the real-world cohort is a significant supplement to NMA. The updated analysis of existing evidence integrates new implications into clinical care that may contribute to achieving more optimal management of mCRC (Morano and Sclafani, 2018).

This study has limitations. First, as for any meta-analysis, an NMA inherits all the limits of the included studies, and caution must be applied while extrapolating the results. Second, an NMA is an indirect comparison that cannot substitute large, well-designed RCTs. Still, when head-to-head comparison studies are lacking, an NMA is an optimal evaluation that combines the available data in the literature. Although the random-effects model was used to minimize the heterogeneity, it cannot be eliminated. Heterogeneity can be due to the period (between 2005 and 2020) and the changes in guidelines and practice, different regimens, different support treatments, and different areas of the world. Third, the indirect comparison between cetuximab- and panitumumab-based treatments was limited as the baseline characteristics between the groups were not similar. Fourth, this analysis was based on summary statistics rather than individual patient data. It might result in some covariates affecting treatment outcomes, especially for patients who received diverse lines of subsequent therapies and interventions for metastasis. We could not estimate the impact of these confounding factors on patient outcomes. Last, the sample of the real-world cohort was small; therefore, large-scale real-world studies or phase IV trials about double-drug regimens plus cetuximab/panitumumab and the triple-drug regimen with bevacizumab in left-sided RAS WT mCRC are needed in the further research. Last, since the triple-drug regimen treatment has not been used widely, so the scale in the real-world cohort is small, further big samples cohort study are needed.

In conclusion, this NMA and real-world data report the ranking of three regimens for therapeutic efficacy in patients with left-sided RAS WT mCRC. The double-drug regimen plus cetuximab/panitumumab appears more effective and safer than the triple-drug regimen with bevacizumab. These results might have implications in selecting the first-line treatment in patients with left-sided RAS WT mCRC. Further trials and cohort analyses on this topic would increase confidence in these results.

## Data Availability

The original contributions presented in the study are included in the article/Supplementary Material, further inquiries can be directed to the corresponding authors.

## Abbreviations

AEs, Adverse events; EGFR, Epidermal growth factor receptor; FP, Luoropyrimidine; HR, Hazard ratio; IV, Intravenous; mCRC, Metastatic colorectal cancer; NMA, Network meta-analysis; ORR, Overall response rate; OR, odds ratio; OS, Overall survival; PFS, Progression-free survival; RCTs, Randomized controlled trials; VEGF, Vascular endothelial growth factor; WT, Wild-type, 5-FU, 5-fluorouracil.

## References

[B1] Aslam S. Emmanuel P. (2010). Formulating a researchable question: A critical step for facilitating good clinical research. Indian J. Sex. Transm. Dis. AIDS 31 (1), 47–50. 10.4103/0253-7184.69003 21808439 PMC3140151

[B2] Assenat E. Desseigne F. Thezenas S. Viret F. Mineur L. Kramar A. (2011). Cetuximab plus FOLFIRINOX (ERBIRINOX) as first-line treatment for unresectable metastatic colorectal cancer: A phase II trial. Oncologist 16 (11), 1557–1564. 10.1634/theoncologist.2011-0141 22016477 PMC3233290

[B3] Bray F. Ferlay J. Soerjomataram I. Siegel R. L. Torre L. A. Jemal A. (2018). Global cancer statistics 2018: GLOBOCAN estimates of incidence and mortality worldwide for 36 cancers in 185 countries. Ca. Cancer J. Clin. 68 (6), 394–424. 10.3322/caac.21492 30207593

[B4] Cremolini C. Antoniotti C. Lonardi S. Aprile G. Bergamo F. Masi G. (2018). Activity and safety of cetuximab plus modified FOLFOXIRI followed by maintenance with cetuximab or bevacizumab for RAS and BRAF wild-type metastatic colorectal cancer: A randomized phase 2 clinical trial. JAMA Oncol. 4 (4), 529–536. 10.1001/jamaoncol.2017.5314 29450468 PMC5885260

[B5] Cremolini C. Loupakis F. Antoniotti C. Lupi C. Sensi E. Lonardi S. (2015). FOLFOXIRI plus bevacizumab versus FOLFIRI plus bevacizumab as first-line treatment of patients with metastatic colorectal cancer: Updated overall survival and molecular subgroup analyses of the open-label, phase 3 TRIBE study. Lancet. Oncol. 16 (13), 1306–1315. 10.1016/S1470-2045(15)00122-9 26338525

[B6] Cremolini C. Marmorino F. Loupakis F. Masi G. Antoniotti C. Salvatore L. (2017). TRIBE-2: A phase III, randomized, open-label, strategy trial in unresectable metastatic colorectal cancer patients by the GONO group. BMC Cancer 17 (1), 408. 10.1186/s12885-017-3360-z 28599628 PMC5466800

[B7] Di Nicolantonio F. Martini M. Molinari F. Sartore-Bianchi A. Arena S. Saletti P. (2008). Wild-type BRAF is required for response to panitumumab or cetuximab in metastatic colorectal cancer. J. Clin. Oncol. 26 (35), 5705–5712. 10.1200/JCO.2008.18.0786 19001320

[B8] Fornaro L. Lonardi S. Masi G. Loupakis F. Bergamo F. Salvatore L. (2013). FOLFOXIRI in combination with panitumumab as first-line treatment in quadruple wild-type (KRAS, NRAS, HRAS, BRAF) metastatic colorectal cancer patients: A phase II trial by the gruppo oncologico nord ovest (GONO). Ann. Oncol. 24 (8), 2062–2067. 10.1093/annonc/mdt165 23666916

[B9] Haller D. G. Catalano P. J. Macdonald J. S. O'Rourke M. A. Frontiera M. S. Jackson D. V. (2005). Phase III study of fluorouracil, leucovorin, and levamisole in high-risk stage II and III colon cancer: Final report of intergroup 0089. J. Clin. Oncol. 23 (34), 8671–8678. 10.1200/JCO.2004.00.5686 16314627

[B10] Heinemann V. von Weikersthal L. F. Decker T. Kiani A. Vehling-Kaiser U. Al-Batran S. E. (2014). FOLFIRI plus cetuximab versus FOLFIRI plus bevacizumab as first-line treatment for patients with metastatic colorectal cancer (FIRE-3): A randomised, open-label, phase 3 trial. Lancet. Oncol. 15 (10), 1065–1075. 10.1016/S1470-2045(14)70330-4 25088940

[B11] Higgins J. P. T. Thomas J. Chandler J. Cumpston M. Li T. Page M. J. (2020). Cochrane handbook for systematic reviews of interventions version 6.1. London: Cochrane Collaboration.

[B12] Hurwitz H. I. Tan B. R. Reeves J. A. Xiong H. Somer B. Lenz H. J. (2019). Phase II randomized trial of sequential or concurrent FOLFOXIRI-bevacizumab versus FOLFOX-bevacizumab for metastatic colorectal cancer (STEAM). Oncologist 24 (7), 921–932. 10.1634/theoncologist.2018-0344 30552157 PMC6656450

[B13] Loupakis F. Cremolini C. Masi G. Lonardi S. Zagonel V. Salvatore L. (2014). Initial therapy with FOLFOXIRI and bevacizumab for metastatic colorectal cancer. N. Engl. J. Med. 371 (17), 1609–1618. 10.1056/NEJMoa1403108 25337750

[B14] Marques R. P. Duarte G. S. Sterrantino C. Pais H. L. Quintela A. Martins A. P. (2017). Triplet (FOLFOXIRI) versus doublet (FOLFOX or FOLFIRI) backbone chemotherapy as first-line treatment of metastatic colorectal cancer: A systematic review and meta-analysis. Crit. Rev. Oncol. Hematol. 118, 54–62. 10.1016/j.critrevonc.2017.08.006 28917269

[B15] Morano F. Sclafani F. (2018). Duration of first-line treatment for metastatic colorectal cancer: Translating the available evidence into general recommendations for routine practice. Crit. Rev. Oncol. Hematol. 131, 53–65. 10.1016/j.critrevonc.2018.08.006 30293706

[B16] NCCN Clinical Practice Guidelines in oncology (2021). Colon cancer. Version 2.2021. Fort Washington: National Comprehensive Cancer Network.

[B17] NCCN Clinical Practice Guidelines in oncology (2020). Rectal cancer. Version 1.2021. Fort Washington: National Comprehensive Cancer Network.

[B18] Nitsche U. Maak M. Schuster T. Kunzli B. Langer R. Slotta-Huspenina J. (2011). Prediction of prognosis is not improved by the seventh and latest edition of the TNM classification for colorectal cancer in a single-center collective. Ann. Surg. 254 (5), 793–800. 10.1097/SLA.0b013e3182369101 22042471

[B19] Nordlinger B. Van Cutsem E. Gruenberger T. Glimelius B. Poston G. Rougier P. (2009). Combination of surgery and chemotherapy and the role of targeted agents in the treatment of patients with colorectal liver metastases: Recommendations from an expert panel. Ann. Oncol. 20 (6), 985–992. 10.1093/annonc/mdn735 19153115

[B20] Petrelli F. Tomasello G. Borgonovo K. Ghidini M. Turati L. Dallera P. (2017). Prognostic survival associated with left-sided vs right-sided colon cancer: A systematic review and meta-analysis. JAMA Oncol. 3 (2), 211–219. 10.1001/jamaoncol.2016.4227 27787550

[B21] Rivera F. Karthaus M. Hecht J. R. Sevilla I. Forget F. Fasola G. (2017). Final analysis of the randomised PEAK trial: Overall survival and tumour responses during first-line treatment with mFOLFOX6 plus either panitumumab or bevacizumab in patients with metastatic colorectal carcinoma. Int. J. Colorectal Dis. 32 (8), 1179–1190. 10.1007/s00384-017-2800-1 28424871 PMC5522523

[B22] Rouse B. Chaimani A. Li T. (2017). Network meta-analysis: An introduction for clinicians. Intern. Emerg. Med. 12 (1), 103–111. 10.1007/s11739-016-1583-7 27913917 PMC5247317

[B23] Sargent D. Shi Q. Yothers G. Van Cutsem E. Cassidy J. Saltz L. (2011). Two or three year disease-free survival (DFS) as a primary end-point in stage III adjuvant colon cancer trials with fluoropyrimidines with or without oxaliplatin or irinotecan: Data from 12, 676 patients from MOSAIC, X-ACT, PETACC-3, C-06, C-07 and C89803. Eur. J. Cancer 47 (7), 990–996. 10.1016/j.ejca.2010.12.015 21257306 PMC3073413

[B24] Selcuk A. A. (2019). A guide for systematic reviews: PRISMA. Turk. Arch. Otorhinolaryngol. 57 (1), 57–58. 10.5152/tao.2019.4058 31049257 PMC6461330

[B25] Sterne J. A. C. Savovic J. Page M. J. Elbers R. G. Blencowe N. S. Boutron I. (2019). RoB 2: A revised tool for assessing risk of bias in randomised trials. Bmj 366, l4898. 10.1136/bmj.l4898 31462531

[B26] Tournigand C. Andre T. Achille E. Lledo G. Flesh M. Mery-Mignard D. (2004). FOLFIRI followed by FOLFOX6 or the reverse sequence in advanced colorectal cancer: A randomized GERCOR study. J. Clin. Oncol. 22 (2), 229–237. 10.1200/JCO.2004.05.113 14657227

[B27] Van Cutsem E. Cervantes A. Adam R. Sobrero A. Van Krieken J. H. Aderka D. (2016). ESMO consensus guidelines for the management of patients with metastatic colorectal cancer. Ann. Oncol. 27 (8), 1386–1422. 10.1093/annonc/mdw235 27380959

[B28] Venook A. P. Niedzwiecki D. Lenz H. J. Innocenti F. Fruth B. Meyerhardt J. A. (2017). Effect of first-line chemotherapy combined with cetuximab or bevacizumab on overall survival in patients with kras wild-type Advanced or metastatic colorectal cancer: A randomized clinical trial. JAMA 317 (23), 2392–2401. 10.1001/jama.2017.7105 28632865 PMC5545896

[B29] Wu C. C. Wang J. H. Lin P. C. Liang C. A. Huang C. Y. Lien H. C. (2020). Tumor sidedness and efficacy of first-line therapy in patients with RAS/BRAF wild-type metastatic colorectal cancer: A network meta-analysis. Crit. Rev. Oncol. Hematol. 145, 102823. 10.1016/j.critrevonc.2019.102823 31783291

