# Supplementary figures and images for: The optimal first-line treatment for patients with left-sided RAS wild-type metastatic colorectal cancer: Double-drug regimen or triple-drug regimen therapy
Source: Front Pharmacol. 2022 Sep 30;13:1015510. doi: 10.3389/fphar.2022.1015510 (PMC9561342; doi:10.3389/fphar.2022.1015510)

A

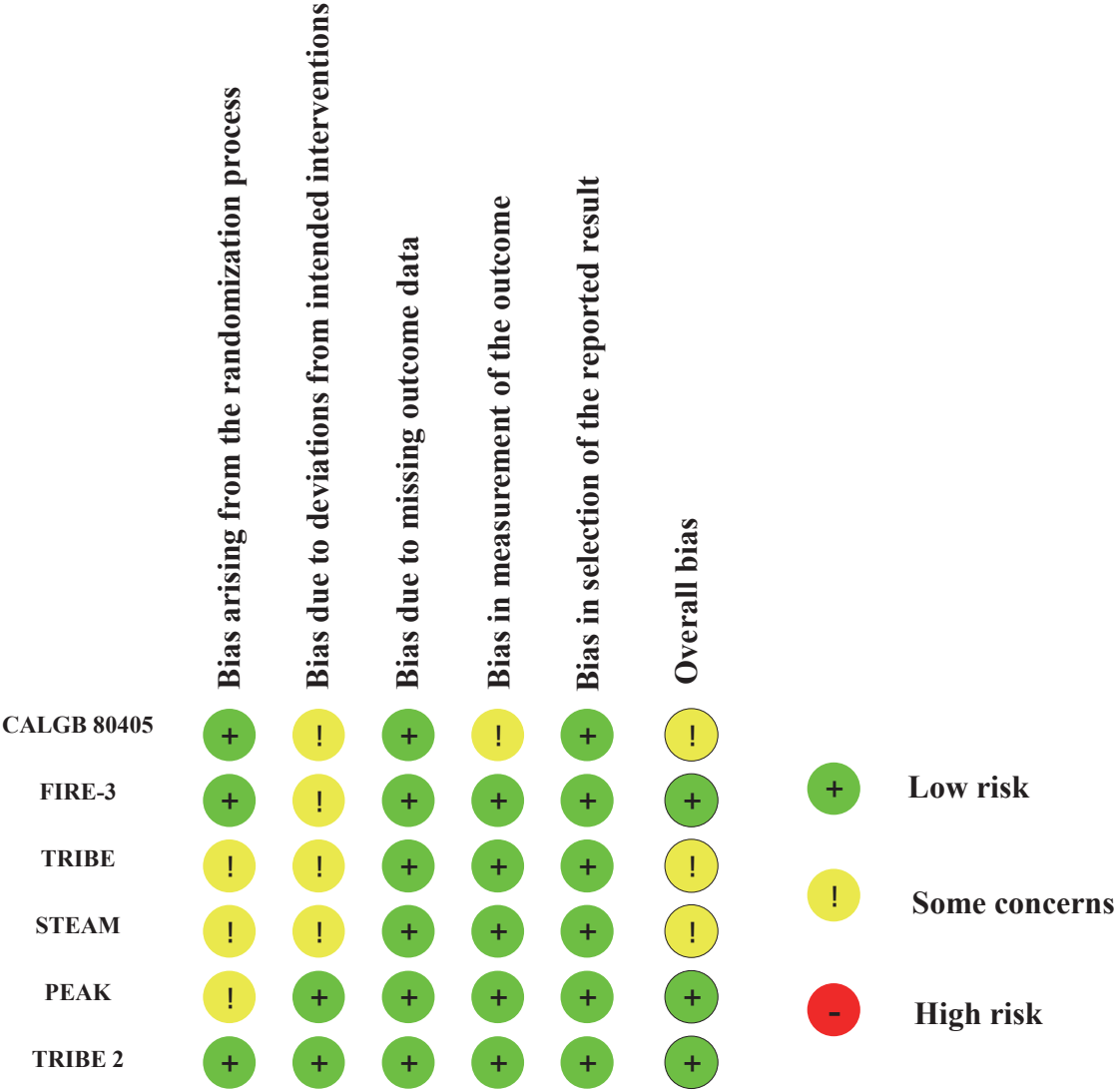

B

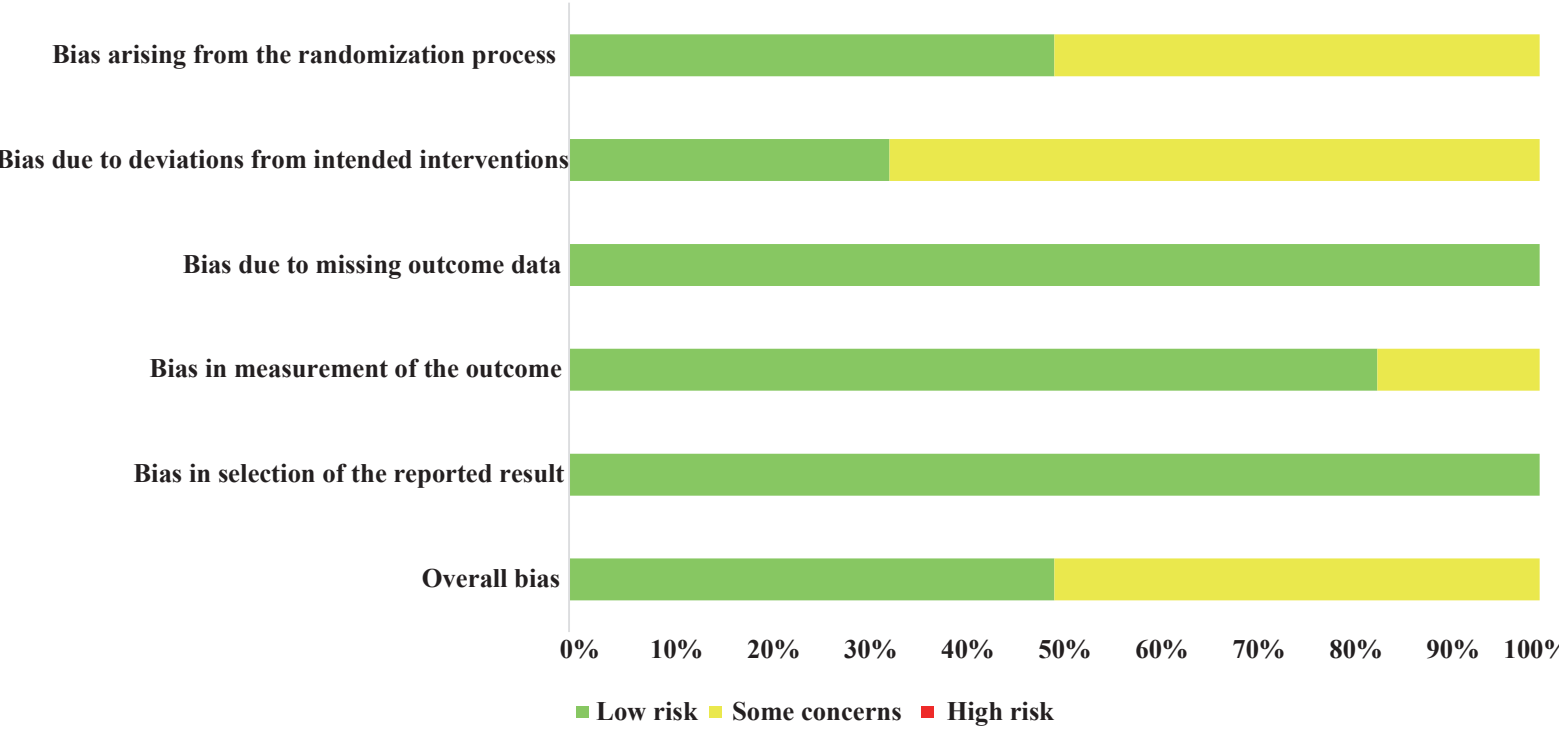

Supplement: Supplementary file 1 [file DataSheet2.PDF]
