# Supplementary material for: The optimal first-line treatment for patients with left-sided RAS wild-type metastatic colorectal cancer: Double-drug regimen or triple-drug regimen therapy
Source: Front Pharmacol. 2022 Sep 30;13:1015510. doi: 10.3389/fphar.2022.1015510 (PMC9561342; doi:10.3389/fphar.2022.1015510)
Supplement: Supplementary file 2 [file DataSheet1.docx]

**Table S1.** Baseline characteristics of the left-sided primary tumors population

| **Year** | **Study name** | **Country** | **Experimental arm** | **Group name A** | **Control arm** | **Group name B** | **Sample size (left-sided)** | **Primary endpoint (s)** | **Secondary endpoint (s)** | **Median Follow-up, months** |
| --- | --- | --- | --- | --- | --- | --- | --- | --- | --- | --- |
| 2014 | FIRE-3 ^25^ | Germany and Australia | FOLFIRI+C | D+C | FOLFIRI+B | D+B | 273 | ORR | PFS, OS | 36 |
| 2017 | CALGB 80405 ^21^ | USA and Canada | FOLFOX/FOLFIRI + C | D+C | FOLFOX/FOLFIRI + B | D+B | 689 | OS | PFS, RR, 60-day mortality | 47.4 |
| 2017 | PEAK ^26^ | Spain, Germany, USA, Belgium, Italy, and Switzerland | FOLFOX6+P | D+P | FOLFOX6+B | D+B | 107 | PFS | OS, ORR, DoR, TTR, Resction rates, Safety | / |
| 2018 | TRIBE ^23^ | Italy | FOLFOXIRI+B | T+B | FOLFIRI+B | D+B | 331 | PFS | RR, OS, Resection rates of metastases | 48.1 |
| 2019 | STEAM ^22^ | USA | FOLFOXIRI+B | T+B | FOLFOX+B | D+B | 105 | ORR, PFS | OS, Safety, Liver resection rate | 22.2 |
| 2020 | TRIBE 2 ^24^ | Italy | FOLFOXIRI+B | T+B | FOLFIRI+B | D+B | 420 | PFS | Safety, ORR, R0 resection rate | 35.9 |
| USA: United States of America; B: bevacizumab; C: cetuximab; D: double-drug; FOLFIRI: fluorouracil, leucovorin, and irinotecan; FOLFOX: fluorouracil, leucovorin, oxaliplatin; FOLFOXIRI: fluorouracil, leucovorin, oxaliplatin, and irinotecan; mFOLFOXIRI: modified FOLFOXIRIORR; P: panitumumab; T: triple-drug. DpR: deepness of response; ORR: overall response rate; OS: overall survival; PFS: progression-free survival; RR, response rate; DoR, Duration of response; TTR, time to response.  NA: not applicable; NR: not reported. | | | | | | | | | | |

Table S2. The Characteristics of the real-world cohort

| Characteristic | No. (%) of Patients | | |
| --- | --- | --- | --- |
|  | D+C | T+B | Total |
| Age (years) |  |  |  |
| ≥50 | 48(57.1%) | 4 (50%) | 52(56.5%) |
| <50 | 36(42.9%) | 4 (50%) | 40(43.5%) |
| Gender |  |  |  |
| Male | 46(54.8%) | 6 (80%) | 52(56.5%) |
| Female | 38(45.2%) | 2 (20%) | 40(43.5%) |
| Colorectal tumor location |  |  |  |
| Left | 31(36.9%） | 1 (12.5%) | 32(34.8%) |
| Right | 17(20.2%) | 5 (62.5%) | 22(23.9%) |
| Multiple | 0 | 1 (12.5%) | 1(1.1%) |
| Rectum | 36(42.9%) | 1 (12.5%) | 37(40.2%) |
